# Familial risk for attention-deficit/hyperactivity disorder and related behaviors in toddlerhood are preliminarily associated with altered frontoparietal development in infancy measured by functional near-infrared spectroscopy

**DOI:** 10.3389/fnins.2026.1919928

**Published:** 2026-10-20

**Authors:** Meghna Krishnamurthy, Judith K. Morgan, Hendrik Santosa, Michelle A. Wilson, Theodore J. Huppert, Heather M. Joseph

**Affiliations:** 1 School of Medicine, University of Pittsburgh, Pittsburgh, PA, United States; 2 Department of Psychiatry, School of Medicine, University of Pittsburgh, Pittsburgh, PA, United States; 3 Department of Psychology, University of Pittsburgh, Pittsburgh, PA, United States; 4 Department of Radiology, School of Medicine, University of Pittsburgh, Pittsburgh, PA, United States; 5 Department of Psychiatry, University of Pittsburgh Medical Center, Pittsburgh, PA, United States; 6 Department of Psychiatry, Stony Brook University, Stony Brook, NY, United States; 7 Department of Pediatrics, School of Medicine, University of Pittsburgh, Pittsburgh, PA, United States

**Keywords:** Attention Deficit and Hyperactivity Disorder (ADHD), frontopariental network, functional near infrared spectrocopy, infant, toddler (MeSH)

## Abstract

**Introduction:**

Attention-Deficit/Hyperactivity Disorder (ADHD) is a common childhood neurodevelopmental disorder associated with impairments across the lifespan. Children with ADHD have been found to have delayed brain maturation. These delays may be present and detectable with functional near-infrared spectroscopy (fNIRS) as early as infancy for children at familial risk for ADHD. The present study examined longitudinal changes in functional connectivity among infants at high and low familial risk for ADHD, and their association with ADHD-related behaviors in toddlerhood.

**Methods:**

Participants were 44 infants, half at high familial risk for ADHD due to parental ADHD, assessed at 8-10 months and 12-14 months of age. fNIRS was used to measure hemodynamic activation in frontal and parietal regions during a low cognitive load task of attention. In toddlerhood, 18-30 months of age, parents rated ADHD-related behaviors using the Child Behavior Checklist.

**Results:**

Across the sample, longitudinal fNIRS analyses revealed increased frontal connectivity with a notable leftward shift between timepoints. High-risk children showed greater increases in frontoparietal connectivity relative to low-risk peers, with divergent trajectories observed in left dorsolateral prefrontal cortex and right parietal connectivity. Greater parent-reported attention symptoms were broadly associated with increased frontoparietal connectivity.

**Discussion:**

Although most findings did not survive corrections for multiple comparisons, these findings suggest that familial ADHD risk and early ADHD-related behaviors may be associated with atypical frontoparietal and frontal network development across the first year of life, highlighting the potential of fNIRS as a tool for identifying early neural markers of ADHD risk that could be used to identify which infants could benefit from early preventative interventions.

## 1 Introduction

Attention-Deficit/Hyperactivity Disorder (ADHD) is one of the most common childhood neurodevelopmental disorders with prevalence rates as high as 10.5% (CAHMI: The Child Adolescent Health Measurement Initiative, 2022). Core symptoms of ADHD include inattention, hyperactivity, and impulsivity (American Psychiatric Association, 2013). Individuals with ADHD are typically identified at school age, well after performance is impaired (Visser et al., 2014), and symptoms and significant impairment in social, educational, occupational, and health domains often persistent across the lifespan (Kosheleff et al., 2023; Sibley et al., 2016). Therefore, there is growing interest in identifying early markers associated with later diagnosis of childhood ADHD that could support referral and development of early intervention programming before 15–24 months, a sensitive period to reduce severity of ADHD symptoms and associated impairment (Nelson et al., 2009).

Offspring born to parents with ADHD are at a 10-fold risk of developing the disorder with heritability estimates as high as 88% (Chen et al., 2017; Larsson et al., 2013). Thus, familial risk cohorts have been helpful for examining early signals of risk for developing childhood ADHD (see Miller et al., 2023). These studies have identified observable neonatal neurodevelopmental immaturity as well as differences in infant behavior (i.e., hyperactive-impulsive behavior) and temperament (i.e., low effortful control and high negative affect) across the first year of life among offspring born to parents with ADHD (Auerbach et al., 2004, 2005; Joseph et al., 2025; Miller et al., 2020). In line with the “maturation lag” hypotheses of ADHD (El-Sayed et al., 2007), these early behavioral signals of familial risk and increased likelihood of ADHD may be expressions of delayed or altered neural development.

Increasingly, cognitive neurodevelopmental research has examined functional neural activity in the frontoparietal regions underlying typically developing attention and emerging executive function in infancy (Rueda, 2024). Attention develops rapidly across the first year of life; it is initially bottom-up and stimulus driven. There is a transition to voluntarily directed, focused attention with the ability to inhibit response to distractors occurring around 9 months of age (Ruff and Rothbart, 2001; Hendry et al., 2016; Posner et al., 2012; Colombo, 2001; Colombo et al., 2004). This change represents nascent executive control over attention, and individual differences in attention at this point of transition are predictive of later executive functioning skills implicated in ADHD (Hendry et al., 2016). Two attention networks involved in top-down control of attention are the dorsal attention network (DAN) and the frontoparietal network. The DAN consists of the frontal eye fields (FEF), intraparietal sulcus, and superior parietal lobule (Rueda et al., 2015) and is involved in voluntary control of attention to space or objects. The frontoparietal network, including the dorsal lateral prefrontal cortex (dlPFC), intraparietal sulcus, and inferior parietal lobule, is responsible for cognitive control of attention through ongoing adjustments to incoming stimuli allowing for sustained attention (Corbetta and Shulman, 2002; Rueda et al., 2015). Efficiency in connectivity between these frontoparietal regions typically improves with age and mediates the development of executive functioning skills (Buss et al., 2014; Buss and Spencer, 2017).

Children with ADHD may show delayed involvement of the prefrontal cortex and less efficiency in connectivity (requiring greater connectivity to achieve sustained attention) between frontoparietal regions across the first year of life, but this possibility has yet to be tested in part due to prior limitations in functional neuroimaging technology. Recent advances in functional neuroimaging technology now allow for the examination of very early frontal activity and connectivity with parietal regions as they are emerging and relate to executive functioning skills (Ellis et al., 2021; Fiske et al., 2025). Functional near-infrared spectroscopy (fNIRS) provides an opportunity to extend research on the neurodevelopmental underpinnings of attention and examine neural signals of risk for ADHD as early as infancy. fNIRS is a non-invasive, low burden, wearable imaging modality that makes it feasible to conduct repeated assessments across the first year of life to examine changes in brain activity. fNIRS uses near-infrared light to measure changes in oxygenated and deoxygenated hemoglobin in cerebral blood as an indirect measure of metabolic activity in cortical neurons. This measure is similar to functional magnetic resonance imaging blood oxygen level-dependent (BOLD) signal with good convergence across modalities (Huppert et al., 2006). Despite having lower spatial resolution, fNIRS has important advantages over fMRI in use with infants including that data is obtained with the infant awake, the equipment is silent eliminating concerns for noise induced hearing loss, and it is less sensitive to motion artifact. Alterations in the frontoparietal neural networks implicated in attention have been found using fNIRS in children with ADHD (Wang et al., 2020; Bian et al., 2025). It is likely that the neural underpinnings of emerging attention precede the onset of ADHD symptoms may be detectable in infancy with some support for differences detected using fNIRS emerging by toddlerhood (1.5–3.5 years old) for children with greater inattention or familial likelihood for ADHD (Joseph et al., 2024; Kerr-German et al., 2022).

The current study leverages longitudinal data of offspring born to parents with and without ADHD. fNIRS data was collected at two time points in infancy, 8–10 and 12–14 months of age, to investigate frontoparietal connectivity across the second half of the first year of life and in relation to familial likelihood for ADHD as well as later ADHD-related behaviors in toddlerhood. Specifically, we aimed to (1) evaluate change in neural functional connectivity over the first year of life; (2) compare neural functional connectivity between infants at high- and low-familial risk for ADHD; and (3) assess the association between neural connectivity during infancy with ADHD-related behaviors in toddlerhood. Across the sample, we expected to see increasing connectivity between dlPFC-DAN at 12–14 months of age as compared to 8–10 months of age. We hypothesized that infants at high risk for ADHD would have greater connectivity between the dlPFC and regions of the DAN (superior and inferior parietal, FEF) necessary to sustain attention during the low cognitive load task. Further, we predict that these patterns of connectivity will predict ADHD-related symptoms in toddlerhood.

## 2 Materials and methods

### 2.1 Participants

Participants were a subsample of infants from the the New(born) Pittsburgh ADHD Risk in Infancy Study (PARIS), a longitudinal, cohort study examining risk for ADHD. Details of participant recruitment can be found in Joseph et al. (2025). Families were excluded if the infant was born prior to 37 weeks gestational age, had a low birth weight (< 5.5 pounds), or was exposed to substances *in utero* at monthly or greater use as determined by mother and father report. Infants with known medical conditions that would interfere with the child's ability to complete the task, including auditory or visual impairment, or conditions known to affect cerebral development or cerebral hemodynamics were excluded. Additional exclusion criteria include parent self-reported history of intellectual disability, schizophrenia, or autism spectrum disorder. All participants with infants 8–10 months of age between 2022 and 2024 were invited to participate in the current study with NIRS data collected at two timepoints (8–10 months and 12–14 months of age). The study was approved by the University of Pittsburgh Institutional Review Board, and parents provided informed consent prior to participation.

The final sample included 44 infant offspring. Originally, 59 infants were recruited; however, 15 participants were removed from the present analysis due to infant fussing/crying (*n* = 8), technical error (*n* = 1), or missing data at time 2 (*n* = 6) resulting in a final sample size of 44. Of the eight infants with missing NIRS data due to fussing/crying, four were from the high-risk group (at least one parent with ADHD) and four were from the low-risk group (neither parent with ADHD). All 44 families provided parent-reported behavioral data in toddlerhood (18.0–29.9 months of age; *M* = 19.5 months).

Participant characteristics for the final sample can be found in Table 1. There were no group differences in demographic characteristics or birth-related risk factors. Overall, infants in both groups had very low rates of birth-related risk factors. Three infants, all from the low-risk group, were admitted to the NICU following birth (two for observation only due to maternal infection during labor and one for jaundice). One low-risk infant had a mother with gestational hypertension, and one high-risk infant had a mother with gestational diabetes.

**Table 1:** Participant characteristics.

	High-risk (*n* = 22) Mean (SD)	Low-risk (*n* = 22)Mean (SD)	*P*
Child age (months)			
Time 1	9.3 (0.8)	9.3 (0.6)	0.795
Time 2	13.1 (0.7)	12.9 (0.5)	0.211
Toddler follow-up	19.7 (2.5)	19.4 (2.7)	0.675
Gestational age at birth (weeks)	39.3 (1.1)	39.4 (0.7)	0.663
Birth weight (oz)	119.5 (15.6)	122.1 (14.6)	0.573
Head circumference at 1month of age (cm)^1^	37.0 (1.0)	37.3 (0.9)	0.438
	***n (%)***	***n (%)***	***P***
Maternal gestational diabetes	1 (4.5)	0 (0.0)	0.312
Maternal gestational hypertension	0 (0.0)	1 (4.5)	0.312
Infant NICU after birth	0 (0.0)	3 (13.6)	0.073
Sex assigned at birth			0.131
Male	13 (59.1)	8 (36.4)	
Female	9 (40.9)	14 (63.6)	
Race and ethnicity			0.736
Asian	0 (0.0)	1 (4.5)	
Black	1 (4.5)	1 (4.5)	
More than one race	2 (9.1)	2 (9.1)	
White, Hispanic/Latino	1 (4.5)	0 (0.0)	
White, not Hispanic/Latino	18 (81.8)	18 (81.8)	
Parent education level			0.472
Less than a bachelor's degree	1 (4.5)	3 (13.6)	
Bachelor's degree	5 (22.7)	3 (13.6)	
Postgraduate degree	16 (72.7)	16 (72.7)	

^1^Head circumference at 1 month of age was available for approximately half of the sample (*n* = 12 low risk, 12 high risk).

Infants received two neurodevelopmental examinations; the NeoNatal Neurobehavioral Scale (NNNS-II; see Joseph et al., 2025) at mean age 6 weeks of age, and the Bayley Scales of Infant and Toddler Development, Third Edition (Bayley-III; Bayley, 2006) at mean age 9 months of age, both administered by trained clinicians. No neonates were identified as clinically concerning or requiring additional workups or referrals on the NNNS-II. No infants scored as developmentally delayed (≥1.5 standard deviations below the mean on any subscale) on the Bayley-III.

### 2.2 Procedures

Participants completed two identical laboratory visits, the first at 8–10 months of age and the second at 12–14 months of age. During each visit, offspring watched *Inscapes* (Vanderwal et al., 2015), a 7-min abstract movie paradigm designed for use in functional imaging studies that captures the focus of participants while minimizing cognitive load as opposed to traditional movies, while their neural activity was simultaneously assessed via NIRS. *Inscapes* has been used for fNIRS with young children (Eng et al., 2025) and has been shown to reduce motion and improve functional connectivity discriminability when compared to typical resting state paradigms (Vanderwal et al., 2015; Hallee et al., 2025). Examiners rated infant attention during the task immediately after administration; infants who were unable to attend to the task were excluded.

Neoprene caps of varying sizes (44 cm−52 cm) were built with four sources and 10 detectors centered on the Fp1 position of the international 10–20 systems covering the prefrontal cortex and posterior parietal region (see Figure 1). To ensure that the locations of our sources and detectors were appropriate for eliciting activation in our regions of interest, we cross-checked our probe using fNIRS Optodes' Location Decider (fOLD) (Zimeo Morais et al., 2018). The distance between sources and detectors was approximately 3 cm but varied slightly on the basis of cap size. Each child's head circumference was measured to determine the appropriate size cap to fit snugly. Optical imaging data were collected with a TechEn CW6 NIRS system (TechEn, Milford, MA, USA) operating in continuous-wave mode with a sampling rate of 20 Hz. The data were measured simultaneously at two wavelengths (690 and 830 nm). Light intensity was automatically adjusted by the system to provide optimal gain.

**Figure 1:**
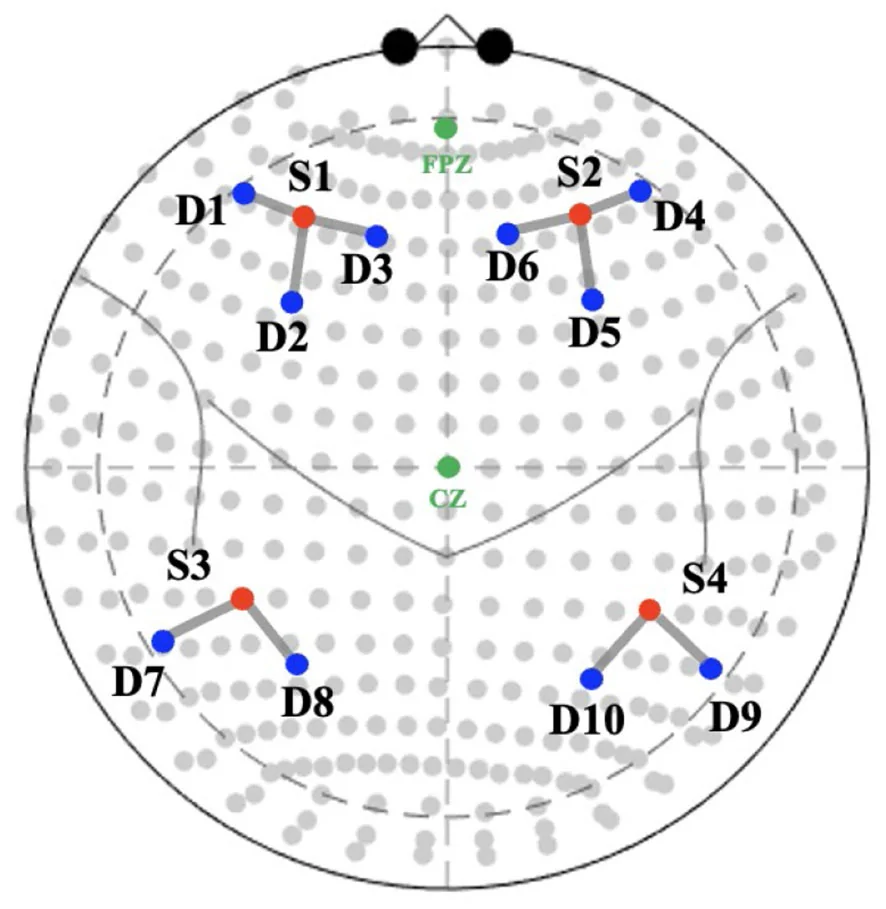
fNIRS probe configuration showing source (S) and detector (D) placement. Detectors were organized across three regions of interest: prefrontal cortex (D1, D3, D4, D6), frontal eye fields (D2, D5), and posterior parietal cortex (D7, D8, D9, D10).

### 2.3 Measures

#### 2.3.1 Conners' adult ADHD diagnostic interview

Parent ADHD status was determined using the semi-structured Conners' Adult ADHD Diagnostic Interview for DSM-IV^TM^ (CAADID; Epstein et al., 2001). It was administered by a research-trained masters- or doctoral-level clinician. Parents were identified as having ADHD if they met DSM-5 criteria for any presentation in both childhood (6+ symptoms) and adulthood (5+ symptoms) with impairment across the lifespan.

#### 2.3.2 Child behavior checklist

Toddler ADHD-related behaviors were assessed using the Child Behavior Checklist for Ages 1½−5 (CBCL; Achenbach and Rescorla, 2000). Both parents were asked to rate their child on 99 different problem behaviors on a 3-point scale (0 = Not True (as far as you know), 2 = Very True or Often True). The DSM-oriented ADHD Problems scale is 6 items (“Can't concentrate, can't pay attention for long”; “Quickly shifts from one activity to another”), with scores of 10 or higher being in the borderline/clinical range. Most (43; 98%) families had reports on toddler ADHD-related behaviors from both parents; one high-risk family had only a mother report (parent with ADHD). For families with both parents reporting, parents' scores were averaged, which yielded good internal consistency in the present sample (α = 0.89).

### 2.4 Preprocessing

The NIRS data were analyzed in Matlab^TM^ (The MathWorks Inc, 2024, Natick, MA, USA; R2024b) using the open-source AnalyzIR toolbox (Santosa et al., 2018). Raw fNIRS signals were first resampled to 1 Hz and converted to changes in optical density. Then, the measured intensity data of the two wavelengths were converted to relative oxy- and deoxyhemoglobin concentration changes using the modified Beer–Lambert law using a differential pathlength factor of 6 (Cope and Delpy, 1988). For resting-state analyses, first-level connectivity was estimated using an autoregressive robust Pearson correlation model. This model accounts for both serially correlated noise arising from systemic physiology signals and heavy-tailed noise associated with motion artifacts. Robust regression with bisquare weighting was used to down-weight outliers (Santosa et al., 2017). This previously validated model has been shown to provide improved sensitivity–specificity characteristics and to reduce false discovery rates caused by serially correlated physiological noise and motion-related artifacts in fNIRS (Huppert, 2016; Santosa et al., 2018).

After the first-level connectivity analysis, we employed a mixed-effects model to examine longitudinal differences in functional connectivity between Visit 1 and Visit 2 across experimental conditions.

For the group-level analysis, we employed linear mixed-effects models to examine the relationships between longitudinal connectivity contrast (Visit 1 vs. Visit 2) and behavioral measures while accounting for repeated observations across participants. Two models were evaluated. The first model included the continuous behavioral variable of ADHD-related behaviors using the CBCL ADHD Problems scale. The second model included a categorical risk-group variable (High vs. Low risk). For both models, random intercepts were included for participant ID to account for repeated observations within subjects and for child sex to account for variability associated with sex difference.

To further assess the robustness of the mixed effects model findings, non-parametric bootstrap analyses were performed using 10,000 iterations. For each iteration, demographic assignments were randomly shuffled across participants while preserving the original data structure and model specification. The mixed-effects models were then re-estimated to generate empirical null distributions of the test statistics. Statistical significance was determined by comparing the observed model estimates (“true connectivity”) against the bootstrap-derived null distributions, providing a non-parametric assessment of the reliability of the connectivity effects. As the bootstrap procedure shuffles demographic variables specific to attention symptoms and familial risk group, parametric mixed effects models were used for the main effect of visit (Aim 1), while bootstrap analyses were applied to risk group and attention symptom comparisons (Aims 2 and 3). In the results, only findings reaching statistical significance at *p* < 0.05 are reported.

### 2.5 Analytic approach

To test the associations between functional connectivity and toddler behavior, edge-wise linear mixed-effects models were used to relate connectivity to CBCL DSM-oriented ADHD scores. Models included child sex and risk status as covariates. Robust estimation was used to reduce sensitivity to outliers. Primary analyses were conducted within timepoint (Visit 1 and Visit 2), and additional contrasts were used to test the association between attentional symptoms and change in connectivity across timepoints (Visit 2 – Visit 1).

All connectivity analyses were corrected for multiple comparisons using false discovery rate (FDR) correction according to the Benjamini–Hochberg procedure (Benjamini and Hochberg, 1995), and we report below adjusted *p*-values (*q*) for all results. All reported results were restricted to oxygenated hemoglobin (HbO–HbO) channel pairs.

## 3 Results

Across Tables 2–4, R represents the estimated connectivity effect (in correlation units) for the specified model contrast, while t represents the corresponding mixed-effects model test statistic evaluating whether that effect differs from zero. The specific contrast represented differs by table, as detailed in each table's legend.

**Table 2:** Functional connectivity change from Visit 1 (8–10 months) to Visit 2 (12–14 months).

Connectivity change from Visit 1 to Visit 2					
	R	t	df	p	q
L dlPFC - L FEF (S1-D1 – S1-D2)	0.146	2.137	42	0.0385	0.866
L dlPFC – R dlPFC (S1-D3 – S2-D4)	0.082	2.451	42	0.0185	0.832
R FEF - R Parietal (S2-D5 – S4-D10)	−0.034	−2.083	42	0.0434	0.866

**Table 3:** Functional connectivity change from Visit 1 to Visit 2 within each familial risk group and group comparison (High vs. Low).

High risk	R	t	df	p	q
L dlPFC – L FEF (S1-D1 – S1-D2)	0.223	2.338	42	0.024	0.363
L dlPFC – R Parietal (S1-D1 – S4-D9)	0.061	2.936	42	0.005	0.242
L FEF – R Parietal (S1-D2 – S4-D9)	0.063	2.622	42	0.012	0.273
L FEF – R FEF (S1-D2 – S2-D5)	−0.153	−2.104	42	0.041	0.466
Low risk	R	t	df	p	q
L dlPFC – R dlPFC(S1-D1 – S2-D4)	0.123	2.262	42	0.029	1.00
L dlPFC – R Parietal (S1-D1 – S4-D9)	−0.049	−2.341	42	0.024	1.00
R FEF – L Parietal (S2-D5 – S3-D8)	0.082	2.074	42	0.044	1.00
High vs. Low	R	t	df	p	q
L dlPFC – R Parietal (S1-D1 – S4-D9)	0.109	3.731	42	0.001	0.025
L FEF – L dlPFC (S1-D2 – S1-D3)	0.302	2.184	42	0.035	0.519
L FEF – R Parietal (S1-D2 – S4-D9)	0.104	3.068	42	0.004	0.085

**Table 4:** Association of functional connectivity change from Visit 1 to Visit 2 with parent-reported CBCL ADHD problem scale.

ADHD-related behaviors					
	R	t	df	p	q
L dlPFC – R parietal (S1-D1 – S4-D9)	0.019	2.818	42	0.010	0.230
L FEF – R dlPFC (S1-D2 – S2-D4)	−0.032	−2.373	42	0.021	0.315
L dlPFC – R parietal (S1-D3 – S4-D10)	0.022	2.805	42	0.003	0.153
R dlPFC – R FEF (S2-D4 – S2-D5)	0.063	2.106	42	0.032	0.358

### 3.1 Neural connectivity change over first year of life

Connectivity changes from Visit 1 (8–10 months) to Visit 2 (12–14 months) were observed for three channel pairs (Figure 2, Table 2). Connectivity between the left dlPFC and left FEF increased from Visit 1 to Visit 2, as did connectivity between the left and right dlPFC. These findings were consistent with hypothesized increases in dlPFC-DAN connectivity across the first year of life. In contrast, connectivity between the right FEF and right parietal region decreased across visits, reflecting a right-lateralized decrease in within-DAN connectivity. Together, these findings are consistent with a strengthening of left frontal network connectivity alongside a reduction in right frontoparietal connectivity across the first year of life, potentially reflecting an early emergence of hemispheric specialization in frontal networks.

**Figure 2:**
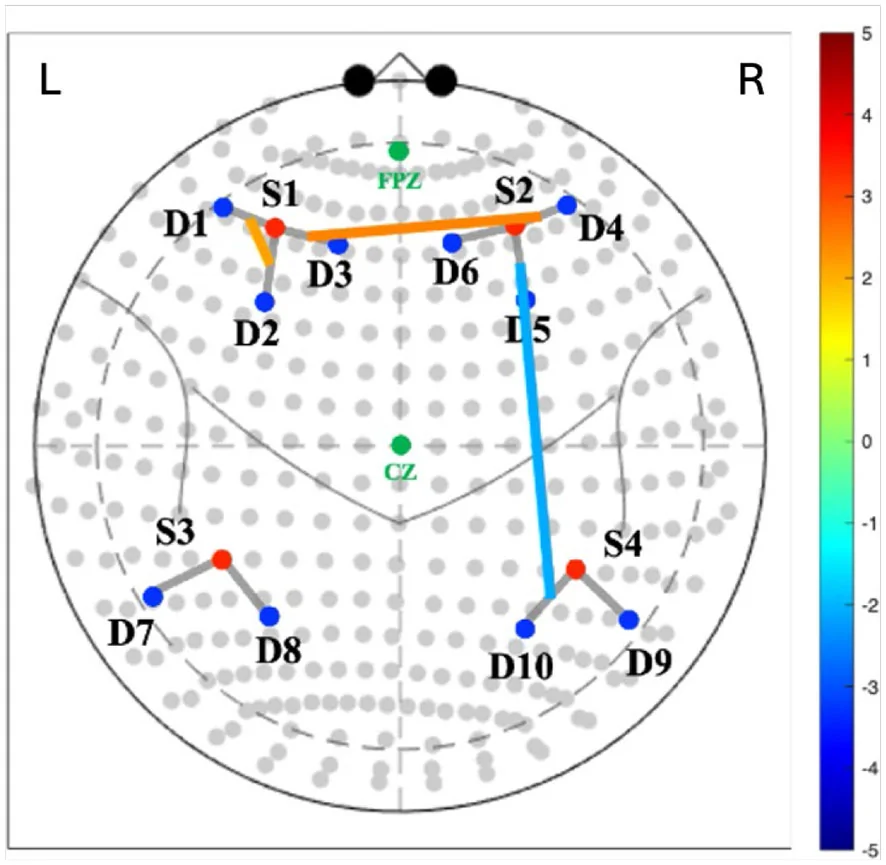
Functional connectivity change from Visit 1 (8–10 months) to Visit 2 (12–14 months of age). Colored lines represent t values between fNIRS channel pairs with significant effects (*p* < 0.05, HbO). D, detector; HbO, oxygenated hemoglobin; S, source.

### 3.2 Group differences in longitudinal connectivity trajectories by ADHD familial risk

At the global level, connectivity did not differ substantially between risk groups or across visits, indicating that group differences were localized to specific frontoparietal connections rather than reflecting a broad, network-wide shift in connectivity strength (Figure 3).

**Figure 3:**
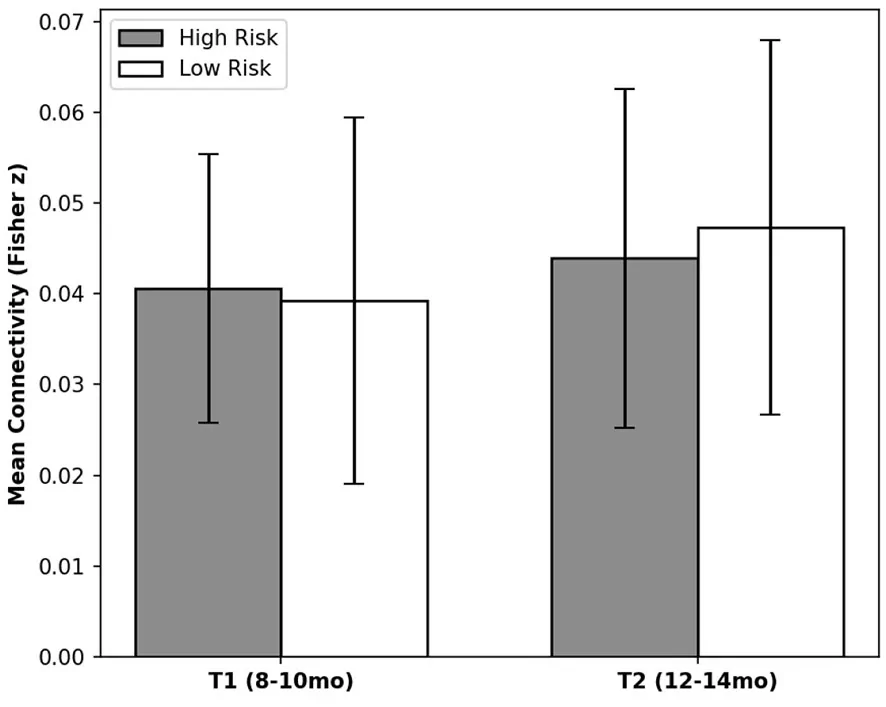
Global network connectivity by familial ADHD risk group and timepoint. Bars represent mean functional connectivity (Fisher z-transformed R) averaged across all 45 tested HbO-HbO channel pairs at Visit 1 (8–10 months) and Visit 2 (12–14 months) for high-risk (gray) and low-risk (white) infants. Error bars represent ± SEM across channel pairs. HbO, oxygenated hemoglobin; SEM, standard error of the mean.

Children at high familial risk for ADHD showed significant increases in connectivity from Visit 1 to Visit 2 between the left dlPFC and components of the DAN, including left frontal eye field and right parietal cortex. An exception to this pattern was the observed decrease in interhemispheric connectivity between bilateral frontal eye fields (Table 3).

Low-risk children showed a significant increase in connectivity within the dlPFC bilaterally. However, a mixed pattern of frontoparietal connectivity was seen with decreased connectivity between the left dlPFC and right parietal cortex but increased connectivity between the right frontal eye field and left parietal cortex (Table 3).

Direct comparison of connectivity change across development between risk groups revealed significantly greater connectivity changes in high- relative to low-risk children (Figure 4). These increases included frontoparietal connections of both the left frontal eye field and left dlPFC with the right parietal cortex, as well as connectivity between the left frontal eye field and left dlPFC (Table 3). Although risk group differences in connectivity between the left frontal eye field and left dlPFC and between the left frontal eye field and right parietal cortex did not survive corrections, the difference in high risk and low risk infants in connectivity between the left dlPFC and right parietal cortex remained statistically significant after FDR correction. Notably, connectivity between the left dlPFC and right parietal area showed divergent developmental trajectories by risk group, with high-risk children showing increased connectivity across visits while low-risk children showed decreased connectivity over the same period, suggesting fundamentally different patterns of frontoparietal network maturation as a function of familial ADHD risk. The largest group difference was observed between the left dlPFC and frontal eye field (R = 0.30), suggesting particularly robust differentiation of local frontal network organization by familial risk status.

**Figure 4:**
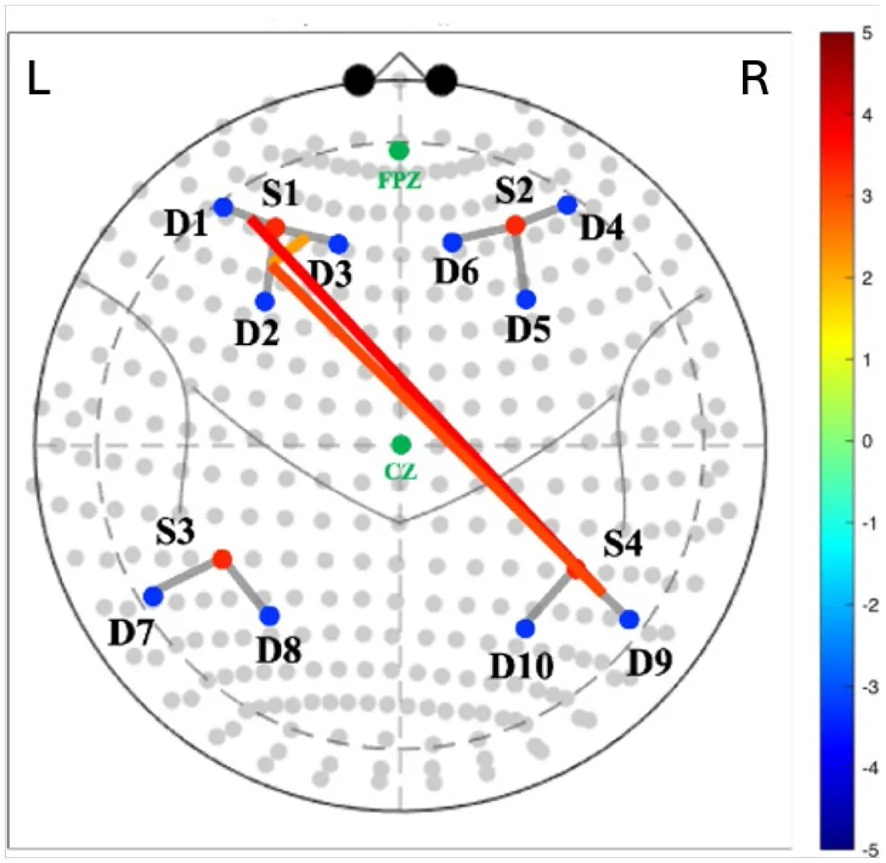
Difference in functional connectivity change across visits between children at high- vs. low-familial risk for ADHD. Colored lines represent t values between fNIRS channel pairs with significant effects (*p* < 0.05, HbO). D, detector; HbO, oxygenated hemoglobin; S, source.

### 3.3 Toddler ADHD-related behaviors by ADHD familial risk

While parents of children in both groups reported few ADHD-related behaviors in their toddlers, toddlers with parental ADHD had greater parent-reported ADHD-related behaviors on the CBCL ADHD Problems scale relative to those without (High risk: *M* = 4.0, *SD* = 2.8, range = 0–11; Low risk: *M* = 1.7, *SD* = 1.5, range = 0–6; *t* = 3.492, *p* = 0.001). Only one child— from the high-risk group— scored in the borderline or clinical range for the ADHD Problems scale.

### 3.4 Associations between infant neural connectivity and toddler ADHD-related behaviors

Greater ADHD-related behaviors were significantly associated with increases in frontoparietal connectivity, including right parietal connections with the left dlPFC (R = 0.019, Figure 5). Right-sided connectivity between the frontal eye field and dlPFC also significantly increased with greater attention symptoms. Notably, greater ADHD-related behaviors were associated with decreased interhemispheric connectivity between the left frontal eye field and right dlPFC across visits (Table 4, Figure 6). These findings did not survive FDR corrections.

**Figure 5:**
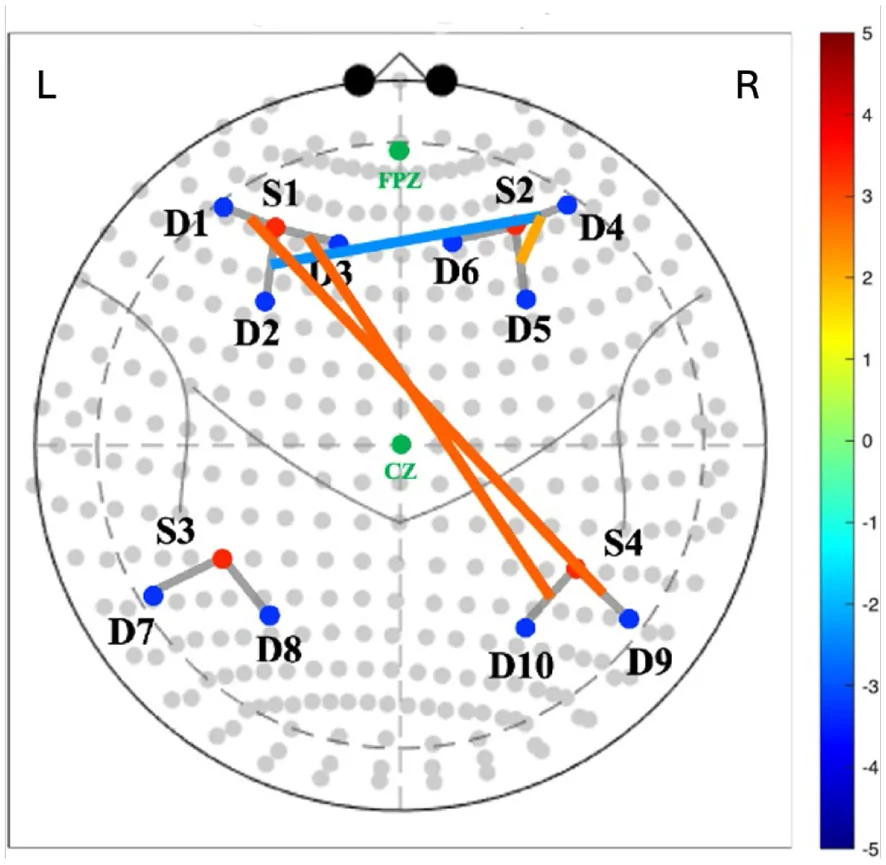
Association of neural functional connectivity change across visits with parent-reported attention symptom severity on the CBCL. Colored lines represent t values between fNIRS channel pairs with significant effects (*p* < 0.05, HbO). CBCL, child behavior checklist; D, detector; HbO, oxygenated hemoglobin; S, source.

**Figure 6:**
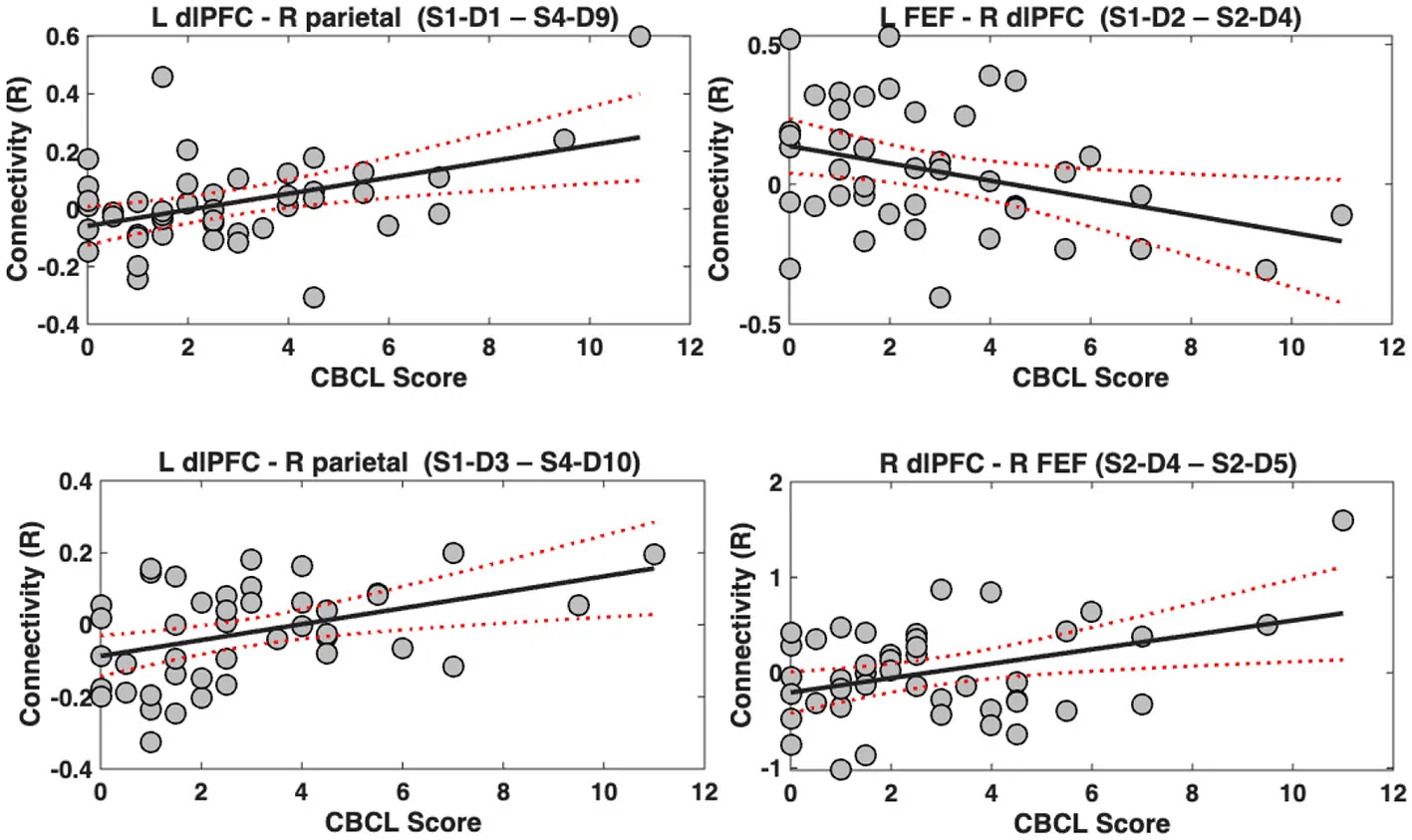
Association of change in neural functional connectivity across visits with parent-reported attention symptom severity on the CBCL. Scatter plots depict connectivity (R) between fNIRS channel pairs with significant effects (*p* < 0.05, HbO) as a function of CBCL DSM-oriented Attention Problems score, with fitted linear trend (black) and 95% confidence interval (red, dotted). CBCL, child behavior checklist; D, detector; dlPFC, dorsolateral prefrontal cortex; FEF, frontal eye field; HbO, oxygenated hemoglobin; L, left; R, right; S, source.

## 4 Discussion

The present findings provide preliminary evidence for developmental changes in frontoparietal connectivity across early life, in addition to differences associated with risk for ADHD. Most notably, high-risk infants showed significantly greater increases in left dlPFC-right parietal connectivity across visits, relative to low risk peers. This finding alone survived FDR correction, while the remaining effects can be interpreted as preliminary. With this caveat in mind, this study's longitudinal analysis revealed increases in connectivity between 8–10 months and 12–14 months of age across frontal eye fields and prefrontal areas, suggesting a strengthening of frontal network organization over the first year of life. We further observed broad increases in frontal and frontoparietal connectivity over the first year of life in association with greater parent-reported ADHD-related behaviors in toddlerhood.

In examining neural functional connectivity changes longitudinally, our findings were consistent with our hypothesis that dlPFC-DAN connectivity increases over the second half of the first year of life. This observed increase was notably localized to the left hemisphere. Our findings support earlier works that have used fMRI to demonstrate increased within-network connection strength for the DAN in neonates across the first year of life (Gao et al., 2012) as well as increased frontal recruitment in conjunction with attentionally demanding tasks (Ellis et al., 2021). We identified a decrease in right-sided DAN intra-network connectivity, which was not mirrored in the left hemisphere across visits. Studies using diffusion MRI and fNIRS in infants have shown the development of leftward asymmetry across the first year of life (Liu et al., 2021, 2022). In infants as young as 3 months, region-level analyses of fNIRS data have shown greater left-sided connectivity that extends to the cortical areas by 6- to 9-months, and greater left-sided efficiency localized around frontal regions (Liu et al., 2022). Our observed hemispheric differences may therefore reflect this early transition from right to left hemispheric dominance as emerging cognitive functions specialize.

Our second objective examined whether longitudinal trajectories of functional connectivity differed between infants at high and low familial risk for ADHD. Broadly consistent with our prediction, high-risk infants showed greater increases in dlPFC-DAN connectivity relative to low-risk peers. The observed connectivity increases in high-risk infants may reflect early compensatory recruitment of dlPFC-DAN networks in response to underlying neurodevelopmental risk. A considerable body of evidence from the literature implicates increased functional connectivity among individuals with ADHD. Functional imaging studies have shown that children with ADHD demonstrate increased connectivity between the prefrontal cortex and the DAN in particular, at ages as young as 7 years old (Wang et al., 2020; Saad et al., 2020). Another study of 8–11 year olds with ADHD performing a flanker task observed greater prefrontal cortex activity, which was positively correlated with inattention symptoms (Suzuki et al., 2017). This earlier work has suggested that greater prefrontal cortex activation in early childhood is necessary to overcome underlying neural pathology and that inefficient mechanisms of network connectivity become difficult to sustain, resulting in inattention.

The most statistically robust evidence for divergent network development between risk groups in this study was the finding that connectivity between the dlPFC and parietal cortex differed significantly between risk groups. The only effect to survive correction for multiple comparisons was the direct comparison between groups, revealing greater L dlPFC – R Parietal connectivity among high-risk infants. Interestingly, high-risk infants showed increased connectivity and low-risk infants showed decreased connectivity between the 8–10 month and 12–14 month timepoints, suggesting qualitatively different patterns of dlPFC-DAN network maturation as a function of familial ADHD risk. The decreasing connectivity observed in the low-risk group may reflect normative patterns for functional brain development during infancy. Bilateral frontoparietal networks have been characterized by a slower developmental trajectory, presenting at an immature form at the end of the first year of life (Gao et al., 2014). Differentiation of networks during this period has been further shown to involve a gradual subdivision into an increasing number of functional modules (Wen et al., 2019). The decline in L dlPFC to R parietal connectivity in low-risk infants may therefore reflect the expected process of network differentiation, whereby initially diffuse long-range connections are refined as more specialized local circuits emerge. In contrast, the increasing connectivity in the high-risk group aligns with the previously discussed compensatory mechanism, whereby greater neural resources are recruited to support emerging attentional capacities in at-risk children. This difference is particularly noteworthy, as it suggests that risk group differences are not simply a matter of degree but may reflect fundamentally different developmental pathways in dlPFC-DAN network organization during the first year of life.

Divergent frontoparietal trajectories between groups may also be interpreted in light of the distinct functional contributions of frontal and parietal cortex to attention.

Electrophysiological evidence has shown that parietal cortex signals salient stimuli earlier than frontal regions in bottom-up attention, whereas frontal regions lead voluntary top-down attentional control (Buschman and Miller, 2007). The first year of life encompasses an expected developmental shift toward greater voluntary attentional control (Rueda et al., 2005), during which parietal-mediated filtering mechanisms become increasingly engaged. Decreased dlPFC-parietal connectivity in low-risk infants over this period may therefore reflect normative refinement of automatic attentional filtering as networks differentiate. In contrast, increased dlPFC-parietal connectivity in high-risk infants may suggest difficulty discerning relevant from irrelevant stimuli, potentially reflecting an early neural signature of the attentional dysregulation characteristic of ADHD. Given that this finding singularly withstands correction for multiple comparisons, it offers the most credible evidence in this study for how familial risk shapes early attentional network development.

Additional connectivity findings with respect to familial risk, while consistent with aforementioned patterns, did not survive correction for multiple comparisons and are discussed here as preliminary. High-risk infants demonstrated a significant decrease in interhemispheric frontal eye field connectivity from 8–10 months to 12–14 months of age. This finding may reflect atypical development of bilateral frontal eye field coordination, which plays a key role in orienting attention (Vernet et al., 2014). Reduced interhemispheric frontal connectivity alongside the broader pattern of increased dlPFC-DAN connectivity observed in high-risk infants may suggest that atypical frontal network development in the context of familial ADHD risk involves both hyperconnectivity within frontoparietal pathways and disrupted coordination between hemispheres.

Our third objective examined whether parent-reported ADHD-related behaviors in toddlerhood were associated with longitudinal changes in functional connectivity in infancy. Greater ADHD-related behaviors in toddlerhood were associated with increased connectivity across three dlPFC-DAN connections, broadly consistent with our prediction of greater dlPFC-DAN connectivity with increasing symptom severity. We also observed a decrease in L FEF–R dlPFC connectivity with greater attention symptom severity, suggesting that interhemispheric frontal coordination may be disrupted in infants with higher attention symptom burden. We consider that this disruption reflects an early vulnerability in neural architecture among infants with greater ADHD-related behaviors, and that the increases in frontoparietal connectivity align with previously described patterns of increased posterior recruitment to sustain attention in these individuals (Cortese et al., 2012; Fassbender and Schweitzer, 2006).

The pattern of increased frontoparietal connectivity with greater attention symptoms parallels the risk group findings discussed above, suggesting that dimensional variation in early ADHD-related symptoms shares a common neural substrate with familial ADHD risk. These findings contribute to a growing body of evidence linking ADHD-related behaviors to frontoparietal hyperconnectivity. Our team previously identified greater frontoparietal connectivity amongst toddlers, 1.5–3 years old, with elevated parent-reported ADHD-related behaviors (Joseph et al., 2024). The findings from the present study suggest that these connectivity changes are detectable even as early as the first year of life. These altered network connectivity patterns appear stable across the developmental time course, as Zhang et al. (2024) further demonstratedincreased frontoparietal connectivity in pre-adolescents with ADHD behavioral symptoms. Collectively, these findings suggest that this hyperconnectivity may reflect compensatory but inefficient recruitment of frontoparietal resources in response to underlying attentional dysregulation- a mechanism that appears to emerge in infancy and persist across development. Together, the convergence between familial risk group and toddler ADHD-related behaviors analyses strengthens the interpretation that increased dlPFC-DAN connectivity represents a shared neural substrate of ADHD vulnerability detectable in infancy.

This study has several noteworthy strengths. The use of a longitudinal design assessing connectivity at two timepoints within the first year of life provides insights into early developmental changes during a critical period of attentional maturation. Among the literature on fNIRS in early childhood, less than a quarter of studies have utilized a longitudinal design, with even fewer targeting populations at risk for ADHD (Su et al., 2023). Examining infant offspring of parents comprehensively diagnosed with ADHD allows investigation of early neural correlates associated with elevated familial risk. Previous research on familial risk for ADHD has relied on self-reported parental ADHD symptoms, or an elevated single symptom of ADHD (e.g., inattention) instead of comprehensive diagnostic interviews to confirm a childhood history and persistent symptoms of ADHD into adulthood. The parent study from which the present sample was recruited is novel in that it examines familial transmission by including semi-structured clinical interview of childhood and adulthood ADHD symptoms and impairment with both biological parents to determine familial risk for ADHD. This rigorous assessment approach increases confidence in the findings.

The findings from this study should be considered in the context of its limitations. Of note, while all findings were significant at an uncorrected threshold of *p* < 0.05, only one survived correction for multiple comparisons, likely reflecting limited statistical power given the modest sample size. These findings should therefore be interpreted with caution and require replication in larger samples before firm conclusions can be drawn. It is also important to consider the inherent instability of functional connectomes during this developmental window, given that the infant brain undergoes rapid development during the first year of life (Dufford et al., 2021). While emerging evidence implicates some replicable patterns in specific functional networks, such as the DAN and DMN, connectome-based identification shows low reliability overall due to dynamic changes at this stage of development (Dufford et al., 2021). This instability may contribute to measurement variability that makes it challenging to identify true effects in our sample. Furthermore, our two assessment timepoints represent age ranges rather than precise ages which may further limit our findings, given the highly dynamic and heterogenous nature of functional connections at this early age range. Our study also stratifies risk status based on familial risk, defining high-risk infants as those born to a parent with ADHD. However, many offspring at familial risk for neurodevelopmental disorders have typical development (Charman et al., 2025). Additionally, while infant and toddler temperament and behavior are predictive of ADHD symptoms and diagnosis in later childhood (Joseph et al., 2023; Miller et al., 2025), ADHD-related behaviors were measured in toddlerhood before a diagnosis of ADHD can be made. This gap between the predisposition to altered network function and the emergence of ADHD symptoms limits our ability to draw conclusions and predict symptom development from infant imaging data.

Future directions should aim to address these limitations with more targeted developmental timepoints and longitudinal follow-up assessing both functional connectivity and ADHD symptoms/diagnosis through mid-childhood. Findings from this study contribute to a growing understanding of how functional connectivity in attentional networks relates to familial risk and early ADHD-related behaviors, offering insights into potential neural markers of ADHD risk that may inform future early intervention efforts.

## Data Availability

The raw data supporting the conclusions of this article will be made available by the authors, on reasonable request and with an executed data use agreement.
